# Signatures of geostrophic turbulence in power spectra and third-order structure function of offshore wind speed fluctuations

**DOI:** 10.1038/s41598-023-40450-9

**Published:** 2023-08-17

**Authors:** So-Kumneth Sim, Joachim Peinke, Philipp Maass

**Affiliations:** 1 Fachbereich Physik, Universität Osnabrück, Barbarastraße 7, 49076 Osnabrück, Germany; 2 Institut für Physik & ForWind, Universität Oldenburg, Küpkersweg 70, 26129 Oldenburg, Germany

**Keywords:** Environmental sciences, Physics

## Abstract

We analyze offshore wind speeds with a time resolution of one second over a long period of 20 months for different heights above the sea level. Energy spectra extending over more than seven decades give a comprehensive picture of wind fluctuations, including intermittency effects at small length scales and synoptic weather phenomena at large scales. The spectra *S*(*f*) show a scaling behavior consistent with three-dimensional turbulence at high frequencies *f*, followed by a regime at lower frequencies, where *fS*(*f*) varies weakly. Lowering the frequency below a crossover frequency $f_\mathrm{\scriptscriptstyle 2D}$, a rapid rise of *fS*(*f*) occurs. An analysis of the third-order structure function $D_3(\tau )$ of wind speed differences for a given time lag $\tau$ shows a rapid change from negative to positive values of $D_3(\tau )$ at $\tau \simeq 1/f_\mathrm{\scriptscriptstyle 2D}$. Remarkably, after applying Taylor’s hypothesis locally, we find the third-order structure function to exhibit a behavior very similar to that obtained previously from aircraft measurements at much higher altitudes in the atmosphere. In particular, the third-order structure function grows linearly with the separation distance for negative $D_3$, and with the third power for positive $D_3$. This allows us to estimate energy and enstrophy dissipation rates for offshore wind. The crossover from negative to positive values occurs at about the same separation distance of 400 km as found from the aircraft measurements, suggesting that this length is independent of the altitude in the atmosphere.

## Introduction

Understanding offshore wind properties is a central problem for forecasting wind power and for estimating wind farm power outputs. Due to the turbulent nature of wind flows in the atmosphere, this is a challenging problem. For three-dimensional (3D) homogeneous isotropic turbulence, a description in terms of Kolmogorov’s theory is possible. Its hallmark is a scaling of kinetic energy spectra with the wavenumber *k* according to a $k^{-5/3}$ law (K41 scaling)^1,2^. This scaling corresponds to a $f^{-5/3}$ scaling in the frequency domain when applying Taylor’s hypothesis^3^. Atmospheric turbulence is, however, different because, apart from seasonal and diurnal influences, scaling features are affected by geometric constraints^4^. An improved understanding of its behavior is one of the grand challenges in wind energy science^5^. For offshore wind, where obstacles such as buildings, trees, and mountains are absent, one could ask whether a generic characterization of wind speed fluctuations over many orders of time or frequency scales is possible.

Spectra of horizontal wind speeds $v=(v_x^2+v_y^2)^{1/2}$, with $v_x$ and $v_y$ being the components parallel to the Earth’s surface, show a deviation from K41 scaling. When a measurement at a small height *h* in the boundary layer is performed, an isotropic and homogeneous inertial (IHI) range of 3D turbulence can no longer be assumed for length scales larger than *h*. In energy (power) spectra *S*(*f*) of wind speeds, the corresponding crossover frequency $f_\mathrm{\scriptscriptstyle IHI}\simeq {\bar{v}}_h/h$, with ${\bar{v}}_h$ the mean wind speed at height *h*, marks the onset of an intermediate regime $f_\mathrm{\scriptscriptstyle 2D}<f<f_\mathrm{\scriptscriptstyle IHI}$ at lower frequencies, where *fS*(*f*) varies weakly. This regime is sometimes referred to as the spectral-gap^6–8^ and its features have been discussed controversially. There is evidence that its properties are dependent on the measurement height *h*^9,10^. Several studies suggest that the spectrum in this regime can show an $f^{-1}$ scaling^11–14^ and different models have been developed to explain such scaling^15–19^. Other fitting functions have been proposed also for describing the behavior^20,21^.

For a long time, it has also been debated whether atmospheric turbulence is characterized by scaling properties of 2D turbulence^22–26^. For isotropic 2D turbulence, the seminal paper by Kraichnan^22^ predicts a regime of $f^{-3}$ scaling to occur at low frequencies as fingerprint of a forward enstrophy cascade, followed by an $f^{-5/3}$ scaling at even lower frequencies due to an inverse energy cascade. For geostrophic winds constrained by rotation and stratification^27,28^, the theory by Charney^29^ predicts that the potential enstrophy is the relevant conserved quantity analogous to 2D turbulence. Geostrophic turbulence behaves like 2D turbulence^29,30^ because of its forward potential enstrophy cascade and conserved total energy^31,32^. The theory of quasi-2D geostrophic turbulence yields one regime of $f^{-3}$ scaling in energy spectra. Nevertheless, energy spectra obtained from aircraft measurements show two scaling regimes with $f^{-5/3}$ and $f^{-3}$ scaling. However, as pointed out by Lindborg^25^, their appearance is not in agreement with the theoretical prediction for isotropic 2D turbulence, because the order of the regimes is reversed. This strongly suggests that the observed $f^{-5/3}$ scaling regime is not due to 2D turbulence. Stratified turbulence^33,34^ and cascades of inertia gravity waves^27,35^ are commonly discussed as possible explanations.

Here we show that spectra *S*(*f*) of offshore wind speeds measured in the North Sea exhibit the commonly observed main features for frequencies $f>f_\mathrm{\scriptscriptstyle 2D}$ as discussed above. For $f<f_\mathrm{\scriptscriptstyle 2D}$, *S*(*f*) rises strongly with decreasing *f* and shows a behavior consistent with the theoretical predictions for quasi-2D geostrophic turbulence in an interval around $10^{-5}\,{\text{Hz}}$. This interval, however, is quite narrow and it is difficult to identify the $f^{-3}$ scaling clearly.

By studying the wind speed fluctuation in the time domain, we provide further evidence that geostrophic turbulence dominates wind speed fluctuations for $f<f_\mathrm{\scriptscriptstyle 2D}$. This evidence comes from analyzing third-order structure functions $D_3(\tau )$, i.e. the third moment of differences between velocities separated by a time $\tau$. The function $D_3(\tau )$ changes sign from negative^1^ to positive values at time lags $\tau \simeq 1/f_\mathrm{\scriptscriptstyle 2D}$, where a positive $D_3(\tau )$ indicates a forward enstrophy cascade^32^. The zero-crossing of $D_3(\tau )$ at $1/f_\mathrm{\scriptscriptstyle 2D}$ is remarkably sharp. By revisiting spectra and third-order structure functions obtained from aircraft measurements^36,37^, we find that frequencies or wavenumbers corresponding to $r_\mathrm{\scriptscriptstyle 2D}$ agree with corresponding crossover frequencies to a $f^{-3}$ scaling regime.

## Data set and data analysis

**Figure 1:**
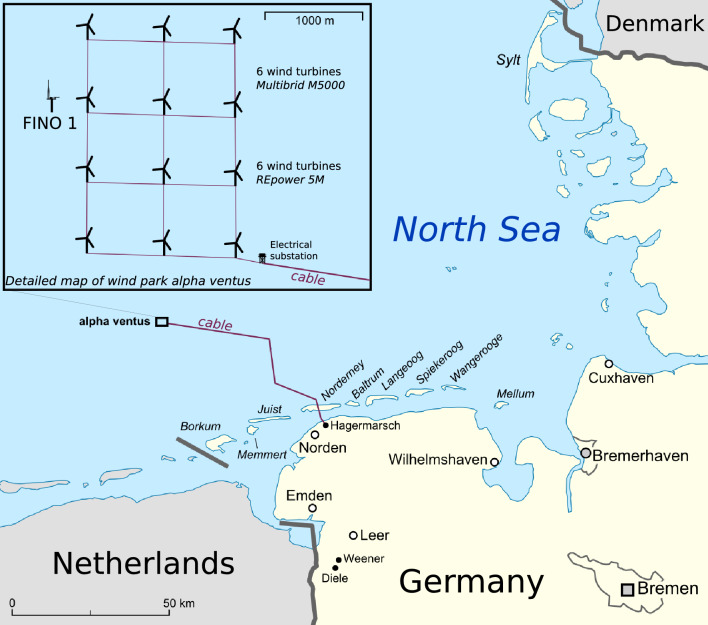
FINO1 platform, located at Alpha Ventus wind farm at Borkum West in the North Sea (54.3$^\text{o}$ N–6.5$^\text{o}$ W). Figure Wind park alpha ventus adapted from Lencer (CC BY-SA 3.0).

Wind speeds were measured at the FINO1 platform in the North Sea, which is located about 45 km north from the island Borkum^38^, see Fig. 1. They were sampled by three-cup anemometers over 20 months, from September 2015 to April 2017, for eight different heights *h* between $30\,{\text{m}}$ and $100\,{\text{m}}$. The time resolution is $\Delta t=1\,{\text{s}}$, yielding time series with $N\cong 5\times 10^7$ speed values for each height (for further details on the data sampling and instrumentation, see FINO - Database information).

**Table 1:** Mean values ${\bar{v}}_h$ and standard deviations $\sigma _h$ of offshore wind speeds for three measurement heights at the FINO1 platform in the North Sea.

*h*	$100\,{\text{m}}$	$60\,{\text{m}}$	$30\,{\text{m}}$
$\bar{v}_h$ $[{{\text{ms}}^{-1}}]$	9.2	8.6	8.2
$\sigma _h$ $[{{\text{ms}}^{-1}}]$	4.8	4.6	4.3
$F_\mathrm{\scriptscriptstyle NaN}$	$0.09\%$	$0.09\%$	$0.35\%$
$\bar{T}_\mathrm{\scriptscriptstyle NaN}$	$12\,{\text{min}}$	$13\,{\text{min}}$	$55\,{\text{min}}$

The number $F_\mathrm{\scriptscriptstyle NaN}$ gives the fraction of NaN entries in the time series that remain after having interpolated single NaN entries. The time $\bar{T}_\mathrm{\scriptscriptstyle NaN}$ is the mean duration of intervals with successive NaN entries after single NaN interpolation.

The time series contain sequences of missing values of different lengths. These “not a number” (NaN) entries require special care in the data analysis, in particular when calculating energy spectra. Single missing values occur typically once a day, i.e. at about every $10^5$th entry in the time series. A single NaN entry at a time $t_\mathrm{\scriptscriptstyle NaN}$ has been replaced by the interpolated value between the two wind speeds at the times $t_\mathrm{\scriptscriptstyle NaN}\pm \Delta t$. In the resulting time series $v_t$ of wind speeds, the fraction $F_\mathrm{\scriptscriptstyle NaN}$ of remaining NaN entries is given in Table 1. Time intervals with successive NaN entries are typically much longer than one second, indicating a temporary failure of the measurement device. The mean duration $\bar{T}_\mathrm{\scriptscriptstyle NaN}$ of the respective intervals is 12 minutes for the measurement heights $h=60\,{\text{m}}$ and $100\,{\text{m}}$, and almost one hour for $h=30\,{\text{m}}$, see Table 1. How we handle these longer time intervals of successive NaN entries is explained below.

Diurnal variations of the offshore wind speeds did not show up as significant patterns in spectra or structure functions and we therefore did not apply a corresponding detrending of the data. We furthermore did neither consider seasonal variations nor changes of meteorologic stability^20^, because we expect them to have only a weak effect on our principal results. Seasonal variations may affect our findings at very long times and corresponding low frequencies only.

Results of our analysis are presented for the three measurements heights $h=30\,{\text{m}}$, $60\,{\text{m}}$, and $100\,{\text{m}}$. The mean ${\bar{v}}_h$ and standard deviation $\sigma _h$ of the wind speeds for these heights are listed in Table 1.

### Energy spectra

For calculating energy spectra, we have used two methods to cope with longer periods of missing values.

In the first method, we determined spectra $S_\alpha (f)$ separately for all time intervals $\alpha$ with existing successive data. These spectra were averaged in bins equally spaced on the logarithmic frequency axis, yielding $S_\text{ave}(f)$.

Specifically, let $\{v_n\}_\alpha =\{v_n^{(\alpha )}\,|\, n=0,\ldots ,N_\alpha -1\}$ be the $\alpha$th sequence of wind speeds without NaN values, $\alpha =1,\ldots ,N_\text{seq}$, where $N_\text{seq}$ is the number of these sequences. The discrete Fourier transform of $\{v_n\}_\alpha$ is

1 $$\begin{aligned} {\hat{v}}_m^{(\alpha )}=\sum _{n=0}^{N_\alpha -1} v_n^{(\alpha )} e^{-2\pi i mn/N_\alpha },\hspace{1em} m=m_\mathrm{\scriptscriptstyle min}^{(\alpha )},m_\mathrm{\scriptscriptstyle min}^{(\alpha )}+1,\ldots ,m_\mathrm{\scriptscriptstyle max}^{(\alpha )}, \end{aligned}$$

where $m_\mathrm{\scriptscriptstyle min}^{(\alpha )}=-\text{int}((N_\alpha -1)/2)$ and $m_\mathrm{\scriptscriptstyle max}^{(\alpha )}=\text{int}(N_\alpha /2)$. The energy spectral density (“energy spectrum”) of $\{v_t\}_\alpha$ at the frequency $f_m^{(\alpha )}=m/T_\alpha$ with $T_\alpha =N_\alpha \Delta t$ is

2 $$\begin{aligned} S_m^{(\alpha )}=S_{-m}^{(\alpha )}=\frac{2\Delta t^2}{T_\alpha }\,|{\hat{v}}_m^{(\alpha )}|^2, \hspace{1em} m=1,\ldots ,m_\mathrm{\scriptscriptstyle max}^{(\alpha )}. \end{aligned}$$

These values $S_m^{(\alpha )}$ for frequencies $f_m^{(\alpha )}$ were averaged in ten bins every decade with equidistant spacing on a logarithmic frequency axis. The left and right border of the *j*th bin are denoted as $f_j^-$ and $f_j^+$, respectively. The averaged energy spectrum in the *j*th bin is

3 $$\begin{aligned} {\bar{S}}_j=\frac{\sum _{\alpha =1}^{N_\text{seq}}\sum _{m=1}^{m_\text{max}^{(\alpha )}} S_m^{(\alpha )} I_j(f_m^{(\alpha )})}{\sum _{\alpha =1}^{N_\text{seq}}\sum _{m=1}^{m_\text{max}^{(\alpha )}} I_j(f_m^{(\alpha )})}, \end{aligned}$$

where $I_j(.)$ is the indicator function of the *j*th bin interval $[f_j^-,f_j^+[$, i.e. $I_j(f)=1$ for $f\in [f_j^-,f_j^+[$ and zero otherwise. The ${\bar{S}}_j$ value gives $S_\text{ave}(f)$ at the frequency $f=(f_j^-f_j^+)^{1/2}$,

4 $$\begin{aligned} S_\text{ave}(f_j)={\bar{S}}_j. \end{aligned}$$

**Figure 2:**
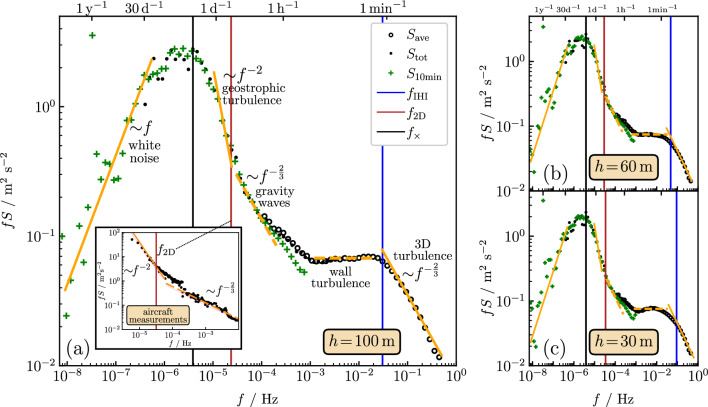
Frequency-weighted energy spectra in a double-logarithmic representation for three different heights (**a**) $h=100\,{\text{m}}$, (**b**) $h=60\,{\text{m}}$, and (**c**) $h=30\,{\text{m}}$. Open circles refer to $fS_\text{ave}(f)$, where $S_\text{ave}(f)$ is obtained by an averaging over all spectra of sub-sequences without missing values (see the description of the energy spectra calculation). Full circles refer to the total spectrum $S_\text{tot}(f)$, where linearly interpolated wind speeds were taken in all intervals of missing data. Green crosses mark spectra calculated from ten minutes averaged wind speeds in the period January 2005–July 2021. The vertical lines separate the various regimes: the blue line at frequency $f_\mathrm{\scriptscriptstyle IHI}=\bar{v}_h/(3h)$ separates the scaling regime of 3D turbulence from the intermediate regime, the red line at frequency $f_\mathrm{\scriptscriptstyle 2D}$ separates the intermediate regime from the regime of quasi-2D geostrophic turbulence, and the black line at frequency $f_\times$ marks the onset of uncorrelated wind speed fluctuations (white noise behavior). The theoretical scaling laws expected in the regimes of geostrophic and 3D turbulence are indicated by orange lines, as well as the white noise behavior at very low frequencies. The inset in (**a**) shows energy spectra obtained from aircraft measurements [extracted from Ref.^36^ and mapped to the frequency domain by applying Taylor’s hypothesis with a mean wind speed $30\,{{\text{ms}}^{-1}}$.].

In the second method, each interval of successive missing values was linearly interpolated between the two wind speed values terminating the interval. The resulting series covers the total time span of 20 months and we calculated its energy spectrum $S_\text{tot}(f)$. This spectrum should agree with $S_\text{ave}(f)$ for frequencies $f\lesssim 1/{\bar{T}}_\mathrm{\scriptscriptstyle NaN}$ and perhaps higher frequencies. Indeed, as shown in Fig. 2 below, the spectra $S_\text{tot}(f)$ (full circles) agree with $S_\text{ave}(f)$ (open circles) in the intermediate frequency range $10^{-4}\,{\text{Hz}}\lesssim f\lesssim 10^{-2}\,{\text{Hz}}$, and even up to frequencies of $10^{-1}\,{\text{Hz}}$ (not shown). This demonstrates that $S_\text{tot}(f)$ is reliable for low frequencies $f<1/{\bar{T}}_\mathrm{\scriptscriptstyle NaN}$.

### Structure functions

In the time domain, characteristic turbulence features can be identified in the scaling behavior of structure functions. The structure function $D_q(\tau )$ of *q*th order at time lag $\tau$ is the *q*th moment of the velocity fluctuation $[v_t - v_{t+\tau }]$,

5 $$\begin{aligned} D_q(\tau )=\left\langle [v_t - v_{t+\tau }]^q\right\rangle _t. \end{aligned}$$

Here, $\langle \ldots \rangle _t$ means an average over all times. We determined the structure functions without replacing missing values by taking the average over all existing pairs $(v_t,v_{t+\tau })$. Knowing $D_q(\tau )$, one can transform this to a function $D_q(r)$ with $r={\bar{v}}_h\tau$, where ${\bar{v}}_h$ is the mean wind speed averaged over the whole time series given in Table 1. This refers to applying Taylor’s hypothesis “globally”.

In a refined analysis, we take into account fluctuations of mean wind speeds on the scale $\tau$. This corresponds to a method sometimes referred to as local Taylor’s hypothesis. Specifically, for a given pair of times *t*, $t+\tau$ we first calculated the average wind speed ${\bar{v}}_{t,t+\tau }$ in the interval $[t,t+\tau [$, ${\bar{v}}_{t,t+\tau }=\sum _{\tau '=0}^{\tau -1} v_{t+\tau '}/\tau$. This gives a distance $r_{t,t+\tau }={\bar{v}}_{t,t+\tau }\tau$ corresponding to Taylor’s hypothesis, i.e. a pair of values $(r,\Delta v(r))=(r_{t,t+\tau },v_t-v_{t+\tau })$. The values $\Delta v(r)^q$ are subsequently averaged in fifty bins every decade with equidistant spacing on the logarithmic *r* axis, yielding $D_q^\text{loc}(r)$, where the superscript indicates the local use of Taylor’s hypothesis. For comparison of $D_q^\text{loc}(r)$ with $D_q(\tau )$, we can transform $D_q^\text{loc}(r)$ back to a function depending on a time lag by using $D_q^\text{loc}(\tau )=D_q^\text{loc}(r/{\bar{v}}_h)$. Differently speaking, applying the local Taylor’s hypothesis amounts to calculating the right-hand side of Eq. (5) for a transformed $\tau '=({\bar{v}}_{t,t+\tau }/{\bar{v}}_h)\tau$.

In our analysis of the wind speed fluctuations in the time domain, we focus on the structure function $D_3(\tau )$ and the kurtosis given by

6 $$\begin{aligned} \kappa (\tau )=\frac{D_4(\tau )}{D_2(\tau )^2}. \end{aligned}$$

When using Taylor’s hypothesis locally, we insert $D_2^\text{loc}(\tau )$ and $D_4^\text{loc}(\tau )$ in this equation, yielding $\kappa ^\text{loc}(\tau )$.

**Figure 3:**
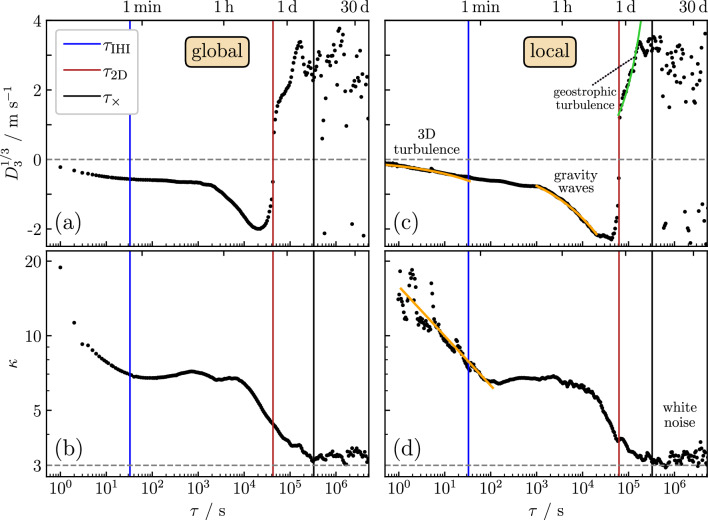
(**a**, **c**) Cubic root of the third-order structure function $D_3(\tau )$, and (**b**, **d**) kurtosis $\kappa (\tau )$ as a function of the time lag $\tau$ for the measurement height $h=100\,{\text{m}}$. Parts (**c**) and (**d**) show the results for $D_3^\text{loc}(\tau )^{1/3}$ and $\kappa ^\text{loc}(\tau )$, when applying the local Taylor’s hypothesis (see description of the data analysis). Vertical blue, red, and black lines correspond to the crossover frequencies in Fig. 2. In (**a**, **c**), the sharp change from negative to positive values at $\tau \gtrsim 1/f_\mathrm{\scriptscriptstyle 2D}$ indicates the transition to quasi-2D geostrophic turbulence. In (**c**), the orange line in the short-time (IHI) regime of 3D turbulence marks the function $-(4/5)\varepsilon _\mathrm{\scriptscriptstyle IHI}{\bar{v}}_h\tau$ with $\varepsilon _\mathrm{\scriptscriptstyle IHI}=3\times 10^{-3}{{\text{m}}^2{\text{s}}^{-3}}$ [cf. Eq. (8)], and the orange line in the regime of turbulence induced by gravity waves marks the function $-2\varepsilon {\bar{v}}_h\tau$ with $\varepsilon =2.5\times 10^{-5}\,{{\text{m}}^{2}{\text{s}}^{-3}}$ [cf. Eq. (10)]. In (**d**), the orange line with slope (-0.2) indicates corrections to K41 scaling corresponding to an intermittency factor $\mu =0.45$. At large time lags $\tau >1/f_\times$, $\kappa (\tau )$ is close to three, corresponding to a Gaussian distribution of velocity fluctuations.

## Results and discussion

Figure 2a shows the frequency-weighted energy spectrum *fS* vs. *f* for the measurement height $h=100\,{\text{m}}$ in a double-logarithmic representation. When comparing the data in Fig. 2a with the corresponding frequency-weighted energy spectra for the other measurement heights in the range $h=30-90\,{\text{m}}$, we have found almost the same functional behavior. This is demonstrated in Fig. 2b and c, where we show the results for $h=60\,{\text{m}}$ and $30\,{\text{m}}$. Similarly, the structure functions $D_q(\tau )$ in the time domain are nearly independent of *h*.

Figure 3a and c show the results for the third-order structure function for $h=100\,{\text{m}}$. We have plotted $D_3(\tau )^{1/3}$ in a semi-logarithmic representation to make changes of the function for small values easier visible. In Fig. 3a, $D_3(\tau )^{1/3}$ is displayed (indicated by “global”), and $D_3^\text{loc}(\tau )^{1/3}$ in Fig. 3c (indicated by “local”). The corresponding results for the kurtosis $\kappa (\tau )$ and $\kappa ^\text{loc}(\tau )$ are shown in Fig. 3b and d. Overall, the results in Fig. 3a and b are similar to that of their counterparts in Fig. 3c and d, although there are differences in detail.

In the following, we first discuss our results for the energy spectra and structure functions in subsections referring to different frequency and respective time regimes. In a final subsection, we compare our findings for the third-order structure function in the crossover regime to quasi-2D geostrophic turbulence with literature results obtained from aircraft measurements.

### IHI regime of 3D isotropic turbulence

Above a frequency

7 $$\begin{aligned} f_\mathrm{\scriptscriptstyle IHI}\sim \frac{{\bar{v}}_h}{h}, \end{aligned}$$

with ${\bar{v}}_h$ the mean wind speed [see Table 1], we see in Fig. 2a the signature of 3D turbulence, i.e., a behavior consistent with the K41 scaling. The border $f_\mathrm{\scriptscriptstyle IHI}$ of this frequency regime is marked by the vertical blue lines in the figure, and the K41 scaling behavior by the solid lines with slope $(-2/3)$.

For the structure functions, the theory of isotropic 3D turbulence^1^ predicts a negative

8 $$\begin{aligned} D_3(r)=-\frac{4}{5}\varepsilon _\mathrm{\scriptscriptstyle IHI}r, \end{aligned}$$

where $\varepsilon _\mathrm{\scriptscriptstyle IHI}$ is the dissipation rate in the isotropic homogeneous inertial range. Taking into account the intermittency corrections to K41 scaling^39^, the kurtosis should scale as

9 $$\begin{aligned} \kappa (\tau )\sim \tau ^{-4\mu /9}, \end{aligned}$$

where $\mu$ is the intermittency factor and quantifies the amplitude of the logarithmic correction in the scaling of the energy dissipation rate with *r*. Values of $\mu$ lie in the range 0.2-0.5^40–42^.

Both $D_3(\tau )$ and $D_3^\text{loc}(\tau )$ in Fig. 3a and c are negative in the regime $\tau \lesssim \tau _\mathrm{\scriptscriptstyle IHI}=1/f_\mathrm{\scriptscriptstyle IHI}$. For the kurtosis shown in Fig. 3b and d, the time $\tau _\mathrm{\scriptscriptstyle IHI}$ marks a crossover time from a regime where $\kappa (\tau )$ decreases to another regime where it is nearly constant. That $\kappa (\tau )$ is much larger than 3 for small $\tau$ reflects fat non-Gaussian tails in the distribution of wind speed fluctuations for short times^43^.

As for the laws (8) and (9), the data in Fig. 3c and d can be well fitted to the respective equations, while this is not the case for the data in Fig. 3a and b. This shows that applying the local Taylor’s hypothesis is needed here.

When fitting $-(4/5)\varepsilon _\mathrm{\scriptscriptstyle IHI}{\bar{v}}_h\tau$ to the data for $D_3^\text{loc}(\tau )$ in Fig. 3b (orange line), we find $\varepsilon _\mathrm{\scriptscriptstyle IHI}=3\times 10^{-3}{{\text{m}}^2{\text{s}}^{-3}}$ for the dissipation rate. This value compares well with results reported in other studies of turbulence in the atmospheric boundary layer^44^. When fitting Eq. (9) to the data for $\kappa ^\text{loc}(\tau )$ in Fig. 3d (orange line), we obtain a slope corresponding to $\mu =0.45$. Deviations from the respective line could be explained by the fact that cup anemometers loose precision for time lags approaching one second.

### Intermediate regime of negative third-order structure function

When *f* becomes smaller than $f_\mathrm{\scriptscriptstyle IHI}$, Fig. 2a–c shows an intermediate regime (IR) where *fS*(*f*) first varies weakly and the K41 scaling is absent. In this regime, the third-order structure function remains negative, see Fig. 3a and c.

On scales $10^{-3}\,{\text{Hz}}\lesssim f<f_\mathrm{\scriptscriptstyle IHI}$ in the IR, *fS*(*f*) is almost constant, or, equivalently, $S(f)\sim f^{-1}$. Also, $D_3(\tau )$ and $\kappa (\tau )$ remain nearly constant in the corresponding time interval. We believe that this behavior reflects turbulent wind patterns strongly influenced by the Earth’s surface, similarly as those found in wall turbulence experiments^45^ for Reynolds numbers larger than $6\times 10^4$ and in atmospheric boundary layers^11,14^. We therefore refer to the regime $10^{-3}\,{\text{Hz}}\lesssim f<f_\mathrm{\scriptscriptstyle IHI}$ as that of “wall turbulence”, see Fig. 2a and denote the lower limit of this regime as $f_\mathrm{\scriptscriptstyle wt}$, i.e. $f_\mathrm{\scriptscriptstyle wt}\simeq 10^{-3}\,{\text{Hz}}$. The scaling $S(f)\sim f^{-1}$ can be reasoned when considering wall turbulence to be governed by attached eddies^46^. Several models have been discussed to explain this scaling^15–19^. An $f^{-1}$ scaling in the energy spectra corresponds to a logarithmic dependence of $D_2(\tau )$ on $\tau$^47^. The second-order structure function follows this logarithmic behavior approximately for times $1/f_\mathrm{\scriptscriptstyle IHI}\lesssim \tau \lesssim 10^3\,{\text{s}}$ (not shown), similarly as it has been found in near-surface atmospheric turbulence on land^48^.

Below $f_\mathrm{\scriptscriptstyle wt}$ in the IR, *fS*(*f*) increases with decreasing *f*. The structure function $D_3(\tau )$ in the corresponding time interval first decreases to larger negative values, and after passing a minimum rapidly rises towards zero. Interestingly, similar features have been seen in the analysis of wind speed data sampled by aircraft. Energy spectra obtained from aircraft measurements are shown in the inset of Fig. 2a. These data were extracted from Ref.^36^ for different wavenumbers and mapped to the frequency domain by applying Taylor’s hypothesis with a mean wind speed $30\,{{\text{ms}}^{-1}}$ typical for the stratosphere. As will be discussed further below, the frequency range $f_\mathrm{\scriptscriptstyle 2D}<f<f_\mathrm{\scriptscriptstyle wt}$ is likely connected to turbulent behavior induced by gravity waves.

### Transition to quasi-2D geostrophic turbulence

The IR regime terminates at a time lag $\tau _\mathrm{\scriptscriptstyle 2D}$, above which $D_3(\tau )$ becomes positive, see Fig. 3a and c. We interpret $f_\mathrm{\scriptscriptstyle 2D}$ as the frequency, below which quasi-2D geostrophic turbulence is governing wind speed fluctuations. According to the theory of geostrophic turbulence^29^, a scaling $fS\sim f^{-2}$ is predicted due to a forward cascade of potential enstrophy^31^, analogous to the enstrophy cascade of ideal isotropic 2D turbulence^22^. Indeed, Fig. 2a–c show a sudden rapid of *fS* increase towards lower *f* for $f\lesssim f_\mathrm{\scriptscriptstyle 2D}$. When *f* is close to $f_\mathrm{\scriptscriptstyle 2D}$, the data approach a line indicating the expected scaling law $fS\sim f^{-2}$. However, the spectral data alone do not provide convincing evidence for a transition to 2D turbulence. This is due to the limited extent of the frequency interval, where the data are consistent with the expected scaling behavior.

Strikingly, the transition becomes very well identifiable in Fig. 3a and c. Third-order structure functions of quasi-2D geostrophic turbulence^32^ are similar to those of 2D turbulence, which are positive in general^49,50^. The third-order structure functions $D_3(\tau )$ in Fig. 3a and c indeed display a very sharp transition from negative to positive values at $\tau =\tau _\mathrm{\scriptscriptstyle 2D}\sim 1/f_\mathrm{\scriptscriptstyle 2D}$.

At the frequency $f=1/{{\text{day}}}$ one could have expected a peak to occur due to diurnal variations. Such a peak has indeed been observed in the early analysis of onshore wind data by Van der Hoven^6^. A diurnal peak does not occur in Fig. 2a–c. We believe that this is because of weaker diurnal temperature variations of oceans compared to land masses. For identifying scaling laws of atmospheric turbulence, this is an advantage as well as the absence of mountains or other heterogeneities on land that can inject long-lived coherent structures.

### 3-day peak and white noise behavior at low frequencies

For $\tau \gtrsim \tau _\times$, $\kappa (\tau )$ in Fig. 3b and d reaches a value $\kappa (\tau )\simeq 3$, reflecting Gaussian distributed wind speed fluctuations. The time $\tau _\times$ has a value of about 3 days and corresponds to a frequency $f_\times =1/\tau _\times$, where *fS*(*f*) in Fig. 2a–c runs through a peak maximum. This peak has been attributed to the motion of low and high pressure areas with linear dimension of about $10^3\,{{\text{km}}}$^6^. If we assume Taylor’s hypothesis to hold even at large time scales of order $\tau _\times$, the corresponding spatial scale $r_\times ={\bar{v}}_h\tau _\times \simeq 3\times 10^3\,{\text{km}}$ agrees with this length scale of low and high pressure areas.

For $r\gtrsim r_\times$, wind speed fluctuations can be expected to become uncorrelated. Accordingly, the energy spectrum should become constant for $f<f_\times$. To test this expectation, one needs very long time series to suppress numerical noise in the spectra. The FINO1 project^38^ also provides ten minutes averaged wind speeds in the long period January 2005 until July 2021. Taking these data, we calculated energy spectra $S_\text{10min}(f)$ with the same method as used for obtaining $S_\text{tot}$. The results are represented by the green crosses in Fig. 2a–c and agree with $S_\text{tot}$ and $S_\text{ave}$ for frequencies below $f_\mathrm{\scriptscriptstyle 2D}$. In the low-frequency regime $f<f_\times$, they indeed show a behavior $fS_\text{10min}\sim f$ of a white noise spectrum. The particular high value of $S_\text{10min}$ at the frequency of 1/year reflects the seasonal cycle of winds at the yearly time scale.

### Comparison of third-order structure function at low altitude with results from aircraft measurements

**Figure 4:**
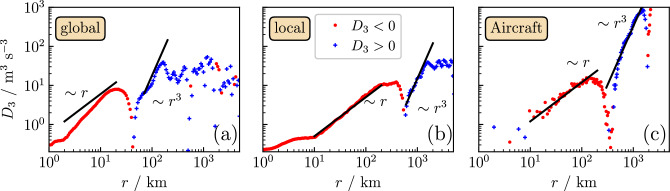
Third-order structure functions as function of separation distance *r* for low altitudes by applying Taylor’s hypothesis with (**a**) a globally averaged velocity, (**b**) with local averages of velocities, and (**c**) for high altitudes taken from aircraft measurements. Blue crosses correspond to positive values of $D_3$ and red dots to negative ones. The theoretical scaling laws expected in the regimes of geostrophic and 3D turbulence are indicated by black lines.

The third-order structure functions obtained from the wind speeds measured at low altitudes of $\sim 100\,{\text{m}}$ above the sea behave very similarly to those obtained from aircraft measurements at very high altitudes of $\sim 10\,{\text{km}}$. For this comparison, we display our results for $D_3$ as a function of the distance *r* in Fig. 4a and b, where for transforming the time lags $\tau$ to distances *r*, we used in (a) the mean wind speed ($r={\bar{v}}_h\tau )$, and in (b) the local Taylor’s hypothesis. The results from aircraft measurements were taken from Cho and Lindborg^37^ and are redrawn in Fig. 4c. The third-order structure functions in Fig. 4a–c show the same overall behavior: a regime of negative $D_3$ at small $r_\mathrm{\scriptscriptstyle 2D}\lesssim \bar{v}_h \tau _\mathrm{\scriptscriptstyle 2D}$ (red symbols) is followed by a regime of positive values at large $r_\mathrm{\scriptscriptstyle 2D}\gtrsim \bar{v}_h \tau _\mathrm{\scriptscriptstyle 2D}$.

A merit of applying the local Taylor’s hypothesis in our analysis becomes clear when comparing the data in Fig. 4a and b. While in Fig. 4b scaling regimes become visible, this is not the case in Fig. 4a. Notably, the results in Fig. 4b show a linear variation of $D_3$ with *r* in the regime of negative $D_3$, and an $r^3$-dependence in the regime of positive $D_3$, see the corresponding lines in the figure. These lines were obtained by least-square fits in the *r* intervals $10\,{\text{km}}<r<200\,{\text{km}}$ and $600\,{\text{km}}<r<1500\,{\text{km}}$.

It is insightful to compare the energy dissipation rate $\varepsilon$ and the enstrophy flux $\eta$ at the different altitudes, which can be extracted from the amplitude factors of the scaling laws. In the regime of linear variation of $D_3$ with *r*, the theory predicts, when incorporating Coriolis forces^51^,

10 $$\begin{aligned} D_3=-2\varepsilon r. \end{aligned}$$

Our analysis yields $\varepsilon =2.5\times 10^{-5}\,{{\text{m}}^{2}{\text{s}}^{-3}}$, which is of similar magnitude as $\varepsilon = 6\times 10^{-5}\,{{\text{m}}^{2}{\text{s}}^{-3}}$ obtained from the aircraft data^51^. For the forward enstrophy cascade, the theory predicts^25,32,51^

11 $$\begin{aligned} D_3(r)=\frac{1}{4}\eta r^3 \end{aligned}$$

Our analysis gives $\eta = 6\times 10^{-17}\,{{\text{s}}^{-3}}$, which is about 20 times smaller than the value $\eta \simeq 1.5\times 10^{-15}\,{{\text{s}}^{-3}}$ reported for the aircraft measurements.

The length scale $r_\mathrm{\scriptscriptstyle 2D} \simeq 500\,{\text{km}}$, where $D_3$ crosses zero, can be estimated. Geostrophic turbulence should arise when rotation and stratification constrain synoptic-scale winds to be nearly horizontal^27,28^. The length scale at which rotation becomes as important as stratification is described by the Rossby deformation radius with $r \simeq 500\,{\text{km}}$ as a standard estimation^34^, which is the same as $r_\mathrm{\scriptscriptstyle 2D}$. A dimensional analysis^30,52,53^ that requires only the enstrophy flux $\eta$ and the energy dissipation rate $\varepsilon$ yields a further estimation of $r_\mathrm{\scriptscriptstyle 2D}$. Assuming $\eta r_\mathrm{\scriptscriptstyle 2D}^3 \sim \varepsilon r_\mathrm{\scriptscriptstyle 2D}$, we find $r_\mathrm{\scriptscriptstyle 2D}\sim \sqrt{\varepsilon /\eta }\simeq 600\,{\text{km}}$ from the data in Fig. 4b, and $\sqrt{\varepsilon /\eta }\simeq 200\,{\text{km}}$ from the aircraft measurements in Fig. 4c. These estimates are of the same order of magnitude.

In the analysis of the aircraft measurements, the regime of linear variation $D_3(r)\sim r$ is related to a scaling regime $S\sim k^{-5/3}$ of corresponding wavenumbers *k* in kinetic energy spectra^54^. Gravity waves are commonly believed to be the physical mechanism leading to the corresponding scaling behaviors with the same functional form as for 3D isotropic turbulence^25,27,34,37,54^. We can ask whether the energy spectra for the wind speeds measured at low altitudes reflect this finding. The frequency interval corresponding to the *r* regime $10\,{\text{km}}<r<100\,{\text{km}}$ is $10^{-4}\,{\text{Hz}}< f < 10^{-3}\,{\text{Hz}}$. In this regime, the local slopes in the double-logarithmic plots in Fig. 2a–c indicate a behavior $fS\sim f^{-2/3}$ (or $S\sim f^{-5/3}$), shown by the orange dashed line in Fig. 2a.

## Conclusions

Our analysis shows that the correlation behavior of offshore wind speed fluctuations at times between a few hours and several days is in agreement with the theory of quasi-2D geostrophic turbulence. While features of this turbulence were seen in previous studies based on aircraft measurements, we have found them here for low altitudes in offshore wind. The third-order structure function in the time domain shows a sharp transition from negative to positive values at a time $\tau _\mathrm{\scriptscriptstyle 2D}$. Transforming the third-order structure function $D_3$ from the temporal to the spatial domain, it is strikingly similar to the aircraft data, if the local Taylor’s hypothesis is used for the transformation. In that case, both the linear variation with the distance in a regime of negative $D_3$ (3D turbulence) and the cubic variation with the distance in a regime of positive $D_3$ (2D geostrophic turbulence) become visible. The transition between negative and positive $D_3$ occurs at about the same length scale $400\,{\text{km}}$ for the offshore wind at a height $100\,{\text{m}}$ and the wind measured by aircraft at a height of about $10\,{\text{km}}$. This strongly suggests that the length scale of the transition to 2D geostrophic turbulence is independent of the altitude.

We have given a comprehensive overview of the spectral behavior of offshore winds covering times from seconds to years. At low frequencies $f\ll f_\times \simeq 1/3\,\text{days}$, a white noise behavior is found, i.e. correlations between wind velocities are not seen in the spectrum *S*(*f*). Around $f_\times$, a peak appears in the frequency-weighted spectrum *fS*(*f*) that can be explained by the motion of low- and high-pressure areas in the troposphere. For $f>f_\times$, the spectral energy decreases with increasing frequency. In a regime $f_\times<f<f_\mathrm{\scriptscriptstyle 2D}=1/\tau _\mathrm{\scriptscriptstyle 2D}$, it decays as $S(f)\sim f^{-3}$ as predicted by the theory of geostrophic turbulence. For higher frequencies $f>f_\mathrm{\scriptscriptstyle 2D}$, results from aircraft measurements^36,54^ show a weaker decay $S(f)\sim f^{-5/3}$, which has been interpreted as resulting from 3D turbulence induced by gravity waves. For the wind measured at a low altitude $h\sim 100\,{\text{m}}$, we find indications of such a regime for *f* close to $f_\mathrm{\scriptscriptstyle 2D}$, but with increasing *f* the weighted spectrum *fS*(*f*) soon becomes flat, before it enters for $f>f_\mathrm{\scriptscriptstyle IHI}$ a regime of 3D isotropic turbulence. The crossover frequency $f_\mathrm{\scriptscriptstyle IHI}$ is about ${\bar{v}}/h$, where ${\bar{v}}$ is the mean wind speed. We believe that the intermediate regime $f_\mathrm{\scriptscriptstyle 2D}<f<f_\mathrm{\scriptscriptstyle IHI}$ has two parts: one at high frequency due to wall turbulence with a behavior $S(f)\sim f^{-1}$, and a second one at higher frequencies, which is influenced by gravity waves. A scaling behavior according to gravity wave induced 3D turbulence, however, becomes clearly visible only at higher altitudes.

Our findings shed new light onto the characterization of wind speed fluctuations from micro- to synoptic scales and beyond. Frequencies of the order of $f_\mathrm{\scriptscriptstyle 2D}$ correspond to mesoscale processes on length scales of 10-$100\,{\text{km}}$. A better understanding of the relation between atmospheric phenomena on these mesoscales and microscales governing air flow around wind turbines and wind power plants, is considered as a grand challenge in wind energy science^5^. This in particular concerns multiscale approaches, where a detailed simulation on microscales has to be connected to coarse-grained approaches on large scales. We believe that our findings on geostrophic 2D turbulence below $f_\mathrm{\scriptscriptstyle 2D}$, the associated scaling of wind speed fluctuations, the indications of gravity-wave induced 3D turbulence close to $f_\mathrm{\scriptscriptstyle 2D}$, and the overall characterization of the different frequency regimes can improve the modeling of offshore wind flows across magnitudes of time scales.

## Data Availability

The wind velocity were measured at the FINO1 platform in the North Sea. The FINO1 project is supported by the German Government through BMWi and PTJ. The database is accessible via https://www.fino1.de/en.
